# Safety and efficacy of a novel 0.5% epinastine topical eyelid cream in allergic conjunctivitis: a phase 3 trial

**DOI:** 10.1007/s10384-024-01108-9

**Published:** 2024-09-11

**Authors:** Hiroshi Fujishima, Jun Shoji

**Affiliations:** 1 Department of Ophthalmology, Tsurumi University School of Dental Medicine, 2-1-3 Tsurumi, Tsurumi-ku, Yokohama-shi, Kanagawa 230-8501 Japan; 2 Division of Ophthalmology, Department of Visual Sciences, Nihon University School of Medicine, Tokyo, Japan

**Keywords:** Allergic conjunctivitis, Conjunctival hyperaemia, Epinastine, Ocular itching, Ophthalmic cream

## Abstract

**Purpose:**

The high prevalence of allergic conjunctivitis in Japan necessitates novel, easy-to-use treatment options for prophylactic use. We evaluated the safety and efficacy of a newly-developed 0.5% epinastine topical eyelid cream to prevent the development of allergic conjunctivitis.

**Study design:**

This was a phase 3, single-centre, double-masked, intra-patient randomised trial in asymptomatic adults (aged 20–65 years) with seasonal allergic conjunctivitis in Japan.

**Methods:**

The left and right eyes of eligible patients were randomised to receive a topical application of either 0.5% epinastine cream (~ 30 mg per dose) to one eye or placebo cream to the other (on the outer skin of the upper and lower eyelids) after a conjunctival antigen challenge (CAC) test. Symptom severity was assessed up to 24 h post-treatment. Primary efficacy endpoints were mean ocular itching and conjunctival hyperaemia severity scores in each eye; safety endpoints included adverse events (AEs) and adverse drug reaction (ADRs).

**Results:**

In total, 30 patients (60 eyes) were included in the study. The 0.5% epinastine topical eyelid cream reduced mean ocular itching scores (difference in least squares means ± standard error, − 1.12 ± 0.214; *p <* 0.0001) and mean conjunctival hyperaemia scores (− 0.54 ± 0.197; *p* = 0.0097) 24 h after treatment versus placebo. The 0.5% epinastine topical eyelid cream was well tolerated, with no AEs or ADRs reported.

**Conclusion:**

With its novel route of administration, 0.5% epinastine topical eyelid cream may be considered a unique, easy-to-use, once-daily treatment option to prevent the onset of seasonal allergic conjunctivitis.

## Introduction

Allergic conjunctivitis refers to a group of disorders caused by perennial and seasonal hypersensitivity, associated with type I hypersensitivity reactions, and accompanied by subjective and objective symptoms [1–3]. The most common type is seasonal allergic conjunctivitis, a non-proliferative disease caused by seasonal exposure to airborne antigens and characterised by conjunctival hyperaemia, itching and ocular discharge [1–3].

The global prevalence of allergic conjunctivitis varies widely between regions but is particularly high in Japan [4], where the prevalence was recently estimated to be 48.7% [5]. In this study, the most common form of allergic conjunctivitis in Japan was seasonal allergic conjunctivitis (estimated prevalence, 45.4%), caused specifically by Japanese cedar and cypress pollen (37.4%) [5]. Although allergic conjunctivitis is rarely associated with structural damage or permanent vision loss, it is a burdensome condition that negatively impacts daily activities, emotional wellbeing and overall quality of life [6].

International guidelines recognise a stepwise approach to managing allergic conjunctivitis [7], ranging from allergen identification and avoidance, nonpharmacological options and pharmacological therapy for patients with more severe allergic conjunctivitis [1, 2, 8–10]. Anti-allergic eye drops are the first-line treatment and include histamine H_1_-receptor antagonists (e.g. levocabastine, emedastine), mast cell stabilisers (e.g. cromolyn, lodoxamide) and dual-acting agents (e.g. epinastine, ketotifen) [1–3, 8, 9]. Tacrolimus or pimecrolimus are available for the treatment of the eyelid skin in atopic dermatitis [7].

In Japan, the recommended dosing frequency for most anti-allergic eye drops is 2–4 times daily; however, many patients with allergic conjunctivitis use these agents on an as-needed basis and only after the onset of symptoms [11, 12]. Eye drop instillation can be challenging, particularly when patients experience visual field loss, feel discomfort, or touch the eye drop bottle to the eye immediately after administration [13–15]. Several studies demonstrate the benefits of preventative use (regular use that is compliant with labelled dosing, regardless of symptoms) versus as-needed use of anti-allergy medications for allergic conjunctivitis, including improved symptomatic relief, inflammatory control and quality of life [12, 16], thereby alleviating current symptoms (if present) and reducing the frequency of future exacerbations.

In addition to repositioning anti-allergy medication as a longer-term treatment strategy for allergic conjunctivitis, there remains an unmet clinical need in Japan for an easy-to-use, once-daily dosing, and/or topical application that facilitates prophylactic use and maximises treatment adherence [17]. Epinastine is a dual H_1_-receptor antagonist and mast cell stabiliser, and previous studies have established that epinastine (0.05%) eye drops are well tolerated in patients with allergic conjunctivitis, and can improve ocular itching and conjunctival hyperaemia symptoms with a duration of action of ≥ 4–8 h [18–21]. Based on the proven effectiveness of epinastine drops in the eye, we postulated their effectiveness as a topical application to the outer skin of the eyelids. Since there are no topical cream formulations developed to employ transdermal absorption through the eyelids for intended action on the conjunctiva, a 0.5% epinastine topical eyelid cream (development code STN1011402) was developed as a treatment option to be applied onto the upper and lower eyelids for the prevention of allergic conjunctivitis. This novel route of administration delivers epinastine through the skin of the eyelid to the conjunctiva, where it exerts its effect [22].

The aim of the present phase 3 study was to evaluate the safety and efficacy of 0.5% epinastine topical eyelid cream in Japanese patients with seasonal allergic conjunctivitis, recruited during non-seasonal periods.

## Materials and methods

### Study design

This was a phase 3, double-masked, intra-patient randomised, placebo-controlled trial of 0.5% epinastine topical eyelid cream in patients with a history of seasonal allergic conjunctivitis. The study was conducted at one clinical site (Kitasato University Kitasato Institute Hospital, Tokyo, Japan) between May 13 and July 12, 2022. The study was conducted in compliance with ethical principles based on the Declaration of Helsinki as revised in 2013, standards set forth in the Japanese Pharmaceuticals and Medical Devices Act and Ministerial Ordinance on Good Clinical Practice. The Institutional Review Board reviewed and approved the study protocol before trial commencement, and all patients provided written informed consent to participate. This study is registered with the Japan Registry of Clinical Trials (JRCT ID: jRCT2031220074, available at https://jrct.niph.go.jp/latest-detail/jRCT2031220074).

### Study population

Eligible patients were asymptomatic adults (aged 20–65 years) with a history of seasonal allergic conjunctivitis. Key exclusion criteria included comorbid inflammatory eye conditions or dry eye disease on the ocular surface or anterior segment, recent intraocular surgery (≤ 90 days before screening) or recent treatment for lacrimal punctum occlusion (≤ 30 days before screening). Patients treated with oral corticosteroids ≤ 28 days before screening, or treated with anti-allergic drugs, H_1_-receptor antagonists or non-steroidal anti-inflammatory drugs ≤ 7 days before screening were also excluded. Additional eligibility criteria for this study are provided in Table 1.

**Table 1:** Study eligibility criteria

**Inclusion criteria**
• Age 20–65 years
• History of allergic conjunctivitis and tested positive for Japanese cedar pollen allergy in a serum antigen-specific IgE antibody test
• No symptoms or signs of ocular itching or conjunctival hyperaemia before antigen challenge in both eyes
• Allergic reaction (ocular itching score ≥ 2 and bulbar conjunctival hyperaemia score ≥ 2) after antigen challenge in both eyes
**Exclusion criteria**
• Inflammatory eye disease (e.g. vernal keratoconjunctivitis, atopic keratoconjunctivitis, blepharitis) or dry eye disease on the ocular surface or anterior segment of the eye
• Ophthalmic conditions that require treatment other than allergic conjunctivitis
• Eyelid abnormalities that may affect assessments in this study (e.g. skin disease, severe ptosis)
• History of intraocular surgery (including laser treatment) ≤ 90 days before the start of the screening period
• Treatment aimed at occluding the punctum (e.g. lacrimal punctal plug insertion, surgical punctal closure) ≤ 30 days before the start of the screening period
• Planned use of a prohibited combination drug or combination therapy during the study period (excluding drugs intended to treat symptoms associated with antigen challenge)
• Use of corticosteroids (excluding oral medications), anti-allergic drugs, H_1_-receptor antagonists, non-steroidal anti-inflammatory drugs, immunosuppressants, vasoconstrictors and/or Chinese herbal drugs indicated for conjunctivitis ≤ 7 days before the start of the screening period (excluding topical skin administration to areas other than the head or face)
• History of oral corticosteroid use ≤ 28 days before the start of the screening period
• History of allergen immunotherapy (e.g. for allergic rhinitis) ≤ 3 years before the start of the screening period
• Requires contact lenses (including therapeutic soft contact lenses) during the study period
• Participated in other clinical trials and/or received study drug ≤ 90 days before the start of the screening period
• Previously participated in a clinical trial of 0.5% epinastine topical eyelid cream (excluding patients who did not receive the study drug)
• Pregnant, lactating, possibly pregnant (including positive pregnancy test), planning to become pregnant during the trial period or unable to use appropriate contraception during the trial period

### Study procedures

#### Screening period

**Fig. 1:**
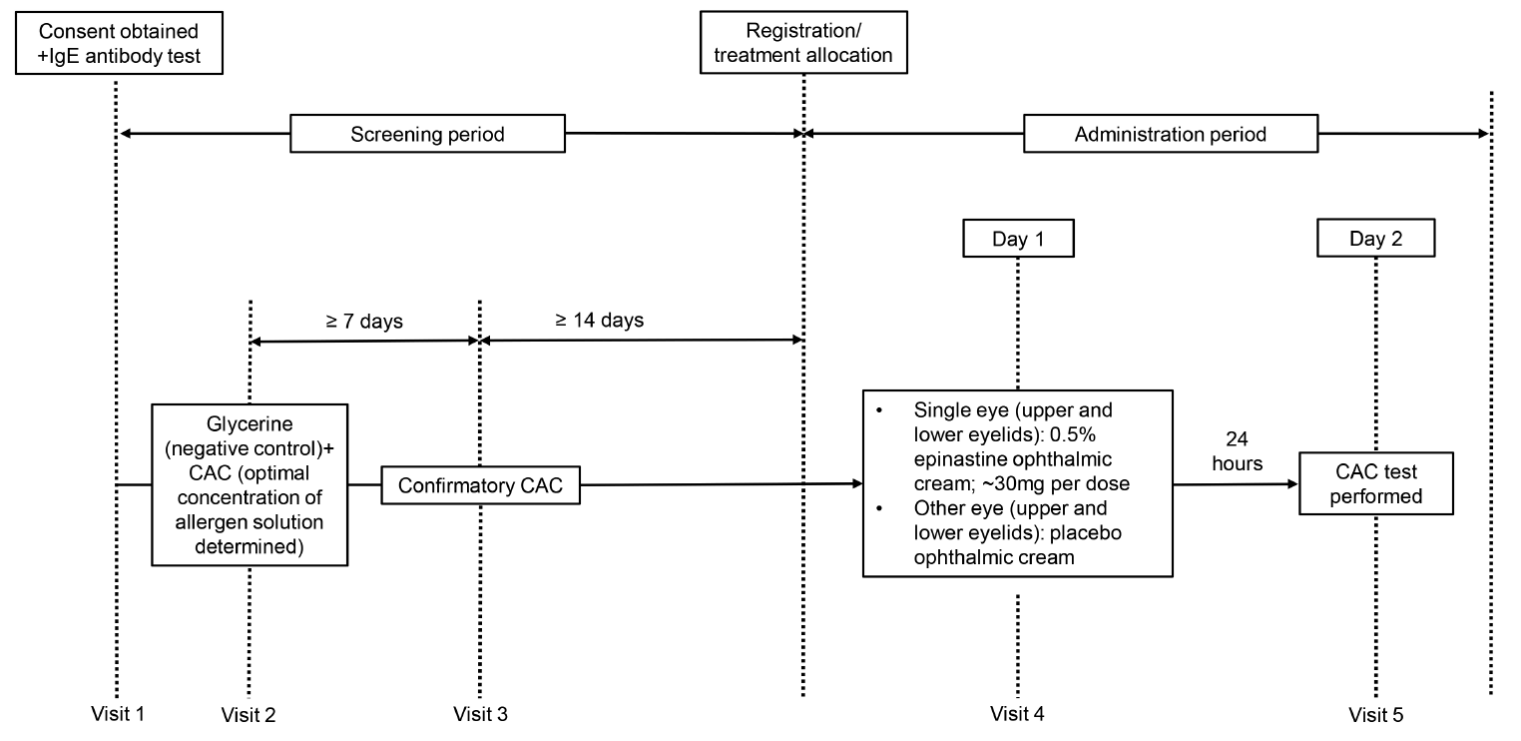
Design of the clinical trial, showing patient visits and treatment periods. CAC, conjunctival allergen challenge; IgE, immunoglobulin E

The study comprised a screening period of ≥ 22 days followed by a treatment period of 2 days (Fig. 1). Eligible patients entered the screening period ≤ 30 days after providing written informed consent; if the screening period began > 30 days after the date of consent, written informed consent was re-collected.

At the start of the screening period (visit 1), eligible patients were tested for Japanese cedar pollen allergy using a serum antigen-specific immunoglobulin E (IgE) antibody test; those who tested negative were excluded from the study. At visit 2 (21 days before the start of the treatment period), conjunctival allergen challenge (CAC) tests were performed to determine the optimal concentration of allergen solution required to trigger allergic conjunctivitis symptoms.

Before the CAC tests, allergen solutions containing Japanese cedar pollen extract (25-fold, 50-fold, 100-fold and 200-fold dilutions) and negative control solutions containing glycerine (25-fold dilution) were prepared (Torii Pharmaceutical Co., Ltd.). All diluted allergen solutions contained glycerine. At visit 2, one drop of negative control solution (without allergen) was instilled in each eye, and patients with a conjunctival inflammatory response were excluded from the study.

At visit 3 (14 days before the start of the treatment period), a confirmatory CAC test was performed using the optimal allergen concentration determined at visit 2. After antigen challenge in both eyes, the severity of ocular itching was assessed at 3, 5 and 10 min, and the severity of bulbar conjunctival hyperaemia was assessed at 5, 10 and 20 min. The optimal allergen concentration was confirmed if ocular itching and bulbar conjunctival hyperaemia severity scores were ≥ 2 in each eye. Throughout the screening period, patients were required to show no signs or symptoms of ocular itching or conjunctival hyperaemia before the CAC tests, and those unable to achieve ocular itching and bulbar conjunctival hyperaemia severity scores ≥ 2 in both eyes after antigen challenge were excluded from the study.

#### Treatment period

On day 1 of the treatment period (visit 4), the left and right eyes of each patient were randomised to receive either 0.5% epinastine topical eyelid cream (approximately 30 mg) [23] or placebo (base cream without epinastine hydrochloride). Eyes were randomly assigned to study cream or placebo using a permuted block method with a block size of 4. Because this was a double-masked study, the study cream and placebo were packaged in identical tubes, and patients and investigators were masked to the treatment allocations for each eye.

At visit 4, study investigators instructed patients to draw a single dose (approximately 1.3 cm of cream as a guide) of 0.5% epinastine topical eyelid cream/placebo cream onto the fingertip to apply onto the periocular area (outer skin of the upper and lower eyelids) of the assigned eye. On day 2 of the treatment period (visit 5; 24 h after application of the study cream and placebo), a CAC test was performed in each eye using the optimal allergen concentration that was determined during the screening period. The severity of ocular itching and bulbar and palpebral conjunctival hyperaemia were assessed after antigen challenge as performed at Visit 3 (Table 2).

**Table 2:** Severity score definitions for allergic conjunctivitis symptoms [20]

Severity score	Definition
**Ocular itching** ^a^	
0	None
1	Intermittent itching
2	Continuous itching
3	Severe itching with desire to scratch, but does not interfere with daily activities
4	Incapacitating itching that interferes with daily activities
**Bulbar conjunctival hyperaemia** ^b^	
0	None
1	Several dilated blood vessels in part of the bulbar conjunctiva
2	Many dilated blood vessels in the entire bulbar conjunctiva
3	Redness of the entire bulbar conjunctiva with indistinguishable single blood vessels
**Palpebral conjunctival hyperaemia** ^b^	
0	None
1	Several dilated blood vessels in part of the palpebral conjunctiva
2	Many dilated blood vessels in the entire palpebral conjunctiva (upper and lower)
3	Redness of the entire palpebral conjunctiva (upper and lower) with indistinguishable single blood vessels

^a^Ocular itching scores were based on patient assessment

^b^Conjunctival hyperaemia scores were based on slit-lamp microscopy findings

### Outcome measures

The primary efficacy endpoints were a 3-time point mean ocular itching score (the average of scores obtained at 3, 5 and 10 min after the antigen challenge) and a 3-time point mean conjunctival hyperaemia score (sum of the bulbar and palpebral conjunctival hyperaemia scores; averaged over 5, 10 and 20 min after the antigen challenge) in each eye on day 2 of the treatment period (24 h after treatment application, in line with the expected once daily administration). Secondary efficacy endpoints included the mean ocular itching, conjunctival hyperaemia, palpebral conjunctival hyperaemia and bulbar conjunctival hyperaemia scores over time during day 2. Treatment effects were assessed by performing CAC tests 24 h after the single-dose application of the study cream and placebo. Safety endpoints included the incidence and severity of adverse events (AEs) and adverse drug reactions (ADRs) during the treatment period, and intraocular pressure (IOP) and funduscopy assessments throughout the screening and treatment periods.

### Statistical analysis

A sample size of 30 patients (60 eyes) was estimated to provide 90% power to detect the superiority of 0.5% epinastine topical eyelid cream versus placebo for the primary endpoints. This was calculated assuming mean ± standard deviation (SD) differences in ocular itching and conjunctival hyperaemia scores of 1.0 ± 0.5 and 1.0 ± 1.6, respectively, paired *t*-tests, type I error rate of 5% and power of 90%.

Baseline demographics and clinical characteristics (measured at visit 1) were based on the intention to treat (ITT) population and summarised using descriptive statistics (means, SDs, medians, ranges, number of patients and/or proportions as appropriate). Efficacy analyses were based on the full analysis set (FAS), defined as all randomised patients who had received ≥ 1 application of the study cream or placebo and for whom efficacy data were available.

Primary efficacy endpoints were summarised using least squares (LS) means, standard errors (SE), mean differences and 95% confidence intervals (CIs). Secondary efficacy endpoints were summarised using means and SDs. The analysis of the primary endpoints was performed on data from the FAS using a linear mixed-effect model, with treatment as the fixed effect and patient as the random effect.

The safety analysis population included all patients who had received ≥ 1 application of study cream or placebo and had available safety data. Safety was assessed through descriptive summaries of AEs, ADRs, IOP and fundoscopy findings by treatment group. AEs and ADRs were coded using the Medical Dictionary for Regulatory Activities’ thesaurus terms and summarised by Preferred Term and System Organ Class. Statistical analyses were performed using SAS version 9.4 or later, and efficacy analyses used a two-tailed significance level of α = 0.05.

## Results

### Study population

**Fig. 2:**
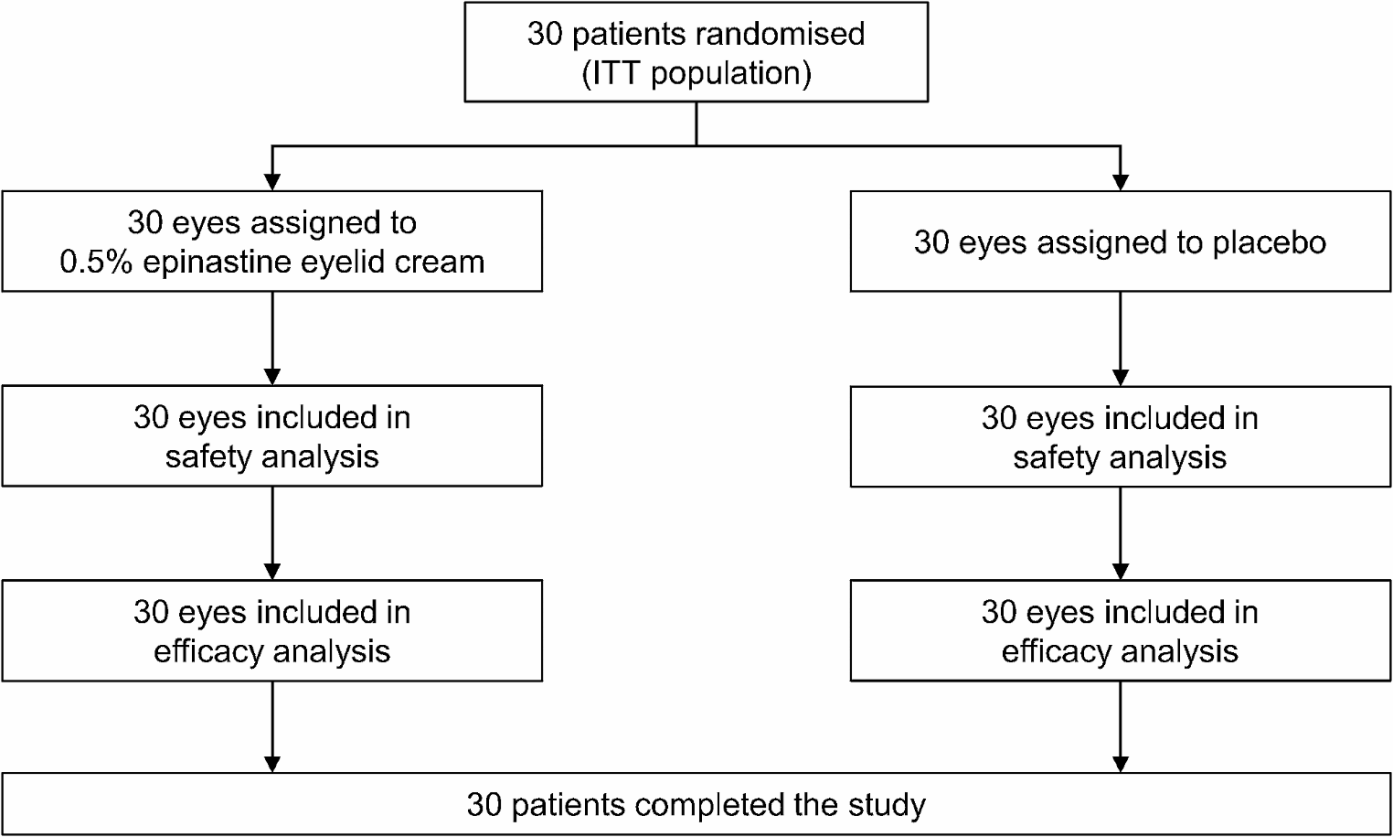
Flow chart of patient populations. ITT, intent-to-treat

In total, 30 patients with a history of seasonal allergic conjunctivitis provided written consent, completed the screening period and were randomised to receive study cream or placebo in each eye (ITT population). All 30 patients received ≥ 1 application of study cream or placebo (Fig. 2). At the start of the screening period, the mean ± SD age of the patients was 43.7 ± 9.9 years, 17/30 (57%) patients were men and by design, all 30 patients tested positive for Japanese cedar pollen allergy (Table 3).

**Table 3:** Baseline patient demographics and clinical characteristics

Baseline characteristic	ITT population (N = 30)
**Age, years** ^a^	
Mean ± SD	43.7 ± 9.9
Median (min, max)	42 (25, 63)
**Age category**,** n (%)**^**a**^	
20–29 years	2 (6.7)
30–39 years	8 (26.7)
40–49 years	12 (40.0)
50–59 years	6 (20.0)
60–64 years	2 (6.7)
**Sex**,** n (%)**^**a**^	
Female	13 (43.3)
Male	17 (56.7)
**Japanese race**,** n (%)**^**a**^	30 (100.0)
**Serum antigen-specific IgE antibody**,** n (%)**^**a**^	
Japanese cedar	30 (100.0)
House dust	8 (26.7)
*Dermatophagoides pteronyssinuts* (dust mite)	8 (26.7)
Common ragweed	3 (10.0)
Japanese cypress	23 (76.7)
**Optimal allergen concentration**,** n (%)**^**b**^	
200-fold dilution	8 (26.7)
100-fold dilution	7 (23.3)
50-fold dilution	10 (33.3)
25-fold dilution	5 (16.7)

**Abbreviations** IgE, immunoglobin E; ITT, intention to treat; SD, standard deviation

^a^Measured at Visit 1 (start of screening period)

^b^Measured at Visit 2 (21 days prior to treatment period) and confirmed at Visit 3 (14 days prior to treatment period)

### Efficacy of 0.5% epinastine topical eyelid cream

On day 2 of the treatment period (24 h after application of study cream and placebo), CAC tests showed that 0.5% epinastine topical eyelid cream was superior versus placebo in reducing mean ocular itching and conjunctival hyperaemia severity scores after antigen challenge (Table 4). The LS mean ± SE ocular itching score (averaged over 3, 5 and 10 min) was significantly reduced in 0.5% epinastine-treated eyes compared with placebo-treated eyes (0.71 ± 0.160 vs. 1.83 ± 0.160; difference − 1.12 ± 0.214; *p* < 0.0001). Similarly, the LS mean ± SE conjunctival hyperaemia score (averaged over 5, 10 and 20 min) was significantly lower in eyes treated with 0.5% epinastine topical eyelid cream versus placebo (2.34 ± 0.278 vs. 2.89 ± 0.278; difference − 0.54 ± 0.197; *p* = 0.0097).

**Table 4:** Mean ocular itching and conjunctival hyperaemia severity scores after antigen challenge on day 2

	0.5% epinastine-treated eyes (n = 30)	Placebo-treated eyes (n = 30)
**Ocular itching score after antigen challenge** ^a^		
LS mean ± SE	0.71 ± 0.160	1.83 ± 0.160
Difference ± SE	−1.12 ± 0.214	
95% CI	−1.559, − 0.686	
*P* value	< 0.0001	
**Conjunctival hyperaemia score after antigen challenge** ^**b**^		
LS mean ± SE	2.34 ± 0.278	2.89 ± 0.278
Difference ± SE	−0.54 ± 0.197	
95% CI	−0.947, − 0.142	
*P* value	0.0097	

**Abbreviations** CI, confidence interval; LS, least squares; SE, standard error

Statistical test was a linear mixed-effects model

^a^Averaged over 3, 5 and 10 min after antigen challenge on day 2

^b^Averaged over 5, 10 and 20 min after antigen challenge on day 2

**Fig. 3:**
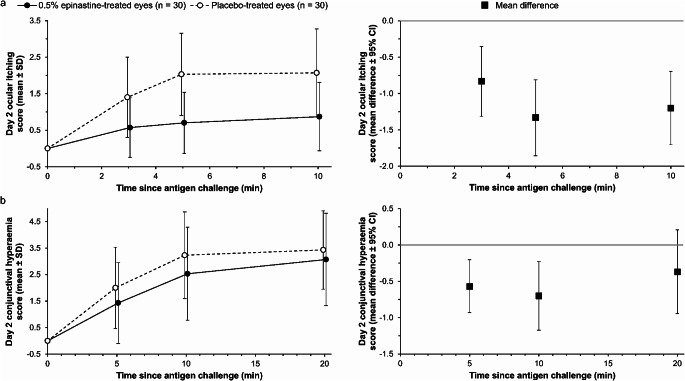
Antigen challenge data showing mean (**a**) ocular itching scores and (**b**) conjunctival hyperaemia scores of epinastine-treated eyes and placebo-treated eyes. The filled circles and empty circles represent the scores measured at each time point for epinastine-treated eyes and placebo-treated eyes, respectively. The graphs to the right in panels (**a**) and (**b**) with the filled squares represent the mean difference between the scores measured at each time point for epinastine-treated eyes and placebo-treated eyes. CI, confidence interval; SD, standard deviation

**Fig. 4:**
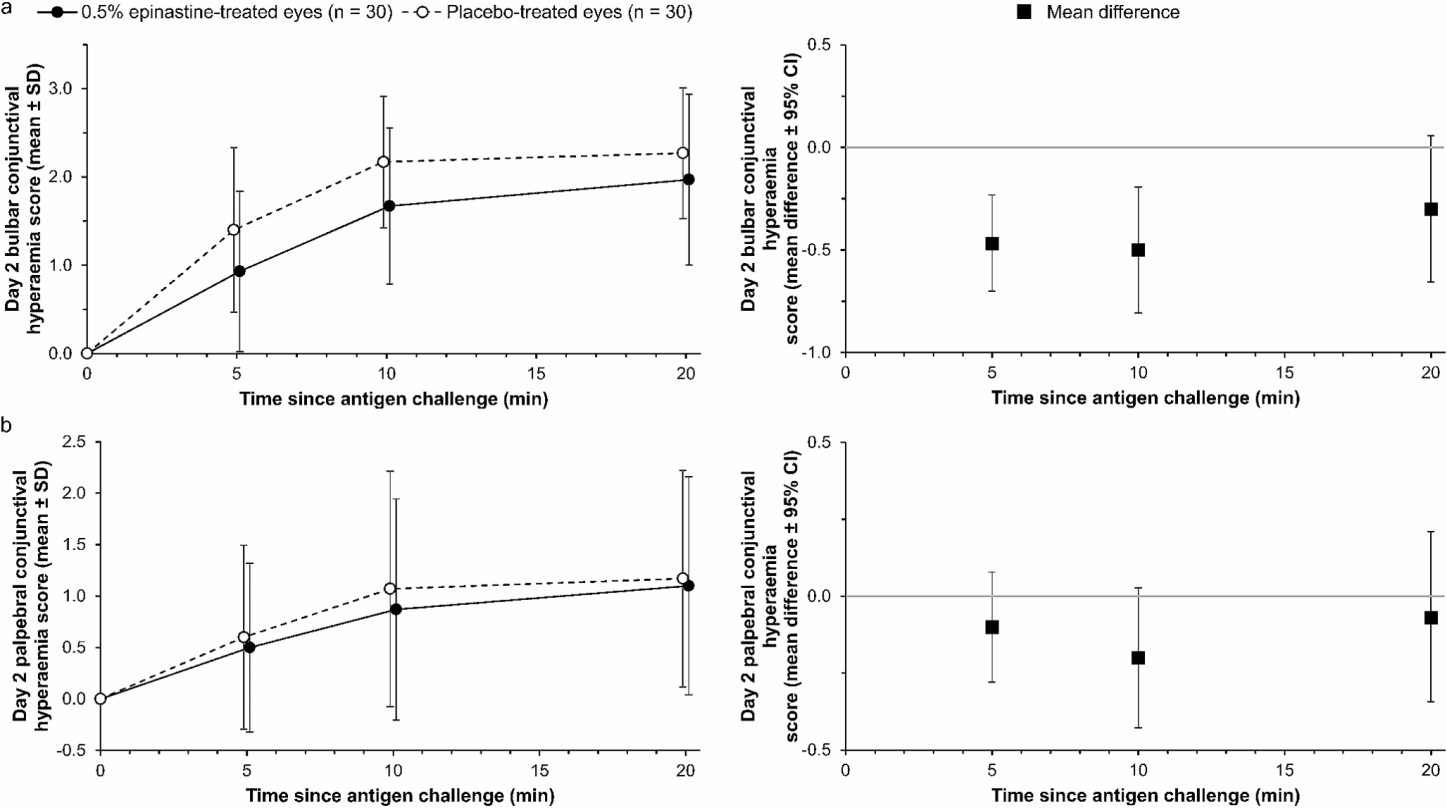
Antigen challenge data showing mean (**a**) bulbar conjunctival hyperaemia scores and (**b**) palpebral conjunctival hyperaemia scores of epinastine-treated eyes and placebo-treated eyes. The filled circles and empty circles represent the scores measured at each time point for epinastine-treated eyes and placebo-treated eyes, respectively. The graphs to the right in panels (**a**) and (**b**) with filled squares represent the mean difference between the scores measured at each time point for epinastine-treated eyes and placebo-treated eyes. CI, confidence interval; SD, standard deviation

In the secondary endpoint analyses, the mean difference in ocular itching scores (Fig. 3a) at 3, 5 and 10 min after antigen challenge on day 2 were − 0.83 (95% CI − 1.315, − 0.352), − 1.33 (95% CI − 1.855, − 0.811) and − 1.20 (95% CI − 1.704, − 0.696), respectively, and the mean difference in conjunctival hyperaemia scores (Fig. 3b) at 5, 10 and 20 min after antigen challenge were − 0.57 (95% CI − 0.929, − 0.204), − 0.70 (95% CI − 1.172, − 0.228) and − 0.37 (95% CI − 0.943, − 0.209), respectively. The mean difference in bulbar conjunctival hyperaemia scores at 5, 10 and 20 min (Fig. 4a) were − 0.47 (95% CI − 0.701, − 0.232), − 0.50 (95% CI − 0.806, − 0.194) and − 0.30 (95% CI − 0.656, 0.056), respectively, and the mean difference in palpebral conjunctival hyperaemia scores at 5, 10 and 20 min (Fig. 4b) were − 0.10 (95% CI − 0.279, 0.079), − 0.20 (95% CI − 0.428, 0.028) and − 0.07 (95% CI − 0.343, 0.210), respectively. When these scores were considered separately, the effect of 0.5% epinastine topical eyelid cream on reducing conjunctival hyperaemia severity was mostly driven by lower bulbar conjunctival hyperaemia scores among 0.5% epinastine-treated versus placebo-treated eyes.

### Safety of 0.5% epinastine topical eyelid cream

In this study, 0.5% epinastine topical eyelid cream was well tolerated by all patients. No AEs or ADRs of any severity were observed during the treatment period. IOP and fundoscopy assessments throughout the study found no clinically relevant changes after application of either 0.5% epinastine topical eyelid cream or placebo.

## Discussion

The benefits demonstrated in this phase 3 study of epinastine 0.5% formulated as a cream for topical application to the outer eyelids are consistent with other published trials of epinastine 0.05% or 0.1% eye drops in patients with allergic conjunctivitis, which show that treatment was well tolerated and significantly reduced ocular itching and conjunctival hyperaemia severity after allergen exposure [18–21, 24]. In an environmental trial, epinastine eye drops were instilled twice daily for 8 weeks [18], while in CAC studies, epinastine was instilled 15 min, 4–8 h before antigen challenge [19–21]. Collectively, these studies found that epinastine eye drops demonstrated sustained treatment effect of ≥ 4–8 h (equivalent to 2–4 times daily dosing) [18–21]. In comparison, 0.5% epinastine topical eyelid cream demonstrated sustained treatment effects 24 h after application to the outer skin of the upper and lower eyelids. These data suggest that the study cream exhibits an extended duration of action to alleviate allergic conjunctivitis symptoms. In addition to the convenience of a once-daily dosage frequency, 0.5% epinastine topical eyelid cream is suitable because of its relative ease of application to the skin of the outer eyelids (rather than the challenge of being instilled into the eye) and can be used proactively as a prophylactic for allergic conjunctivitis.

In this study, the efficacy of 0.5% epinastine topical eyelid cream was assessed using CAC tests, developed as an alternative to, but not a complete substitute for, environmental studies of allergic conjunctivitis [13, 25]. Advantages of CAC tests over environmental studies include the ability to reproduce the signs and symptoms of allergic conjunctivitis in a controlled setting, the transient nature of symptoms after antigen challenge and the level of internal control provided by bilateral administration of study drugs and comparator/placebo [13, 25]. On the other hand, environmental trials allow the effectiveness of ocular anti-allergy agents to be studied in a setting that most accurately reflects real-world clinical practice [13, 25]. In support of the results of the present study, once-daily 0.5% epinastine topical eyelid cream has shown sustained efficacy when administered for 8 weeks in patients with allergic conjunctivitis, with significant reductions in ocular pruritus and conjunctival hyperaemia scores observed from Week 1 of administration and no safety concerns identified. A 0.1% epinastine eye drops formulation (instilled twice daily) was used as a reference; similar efficacy and safety were observed with both formulations, and patients considered 0.5% epinastine topical eyelid cream easy to use and apply and less burdensome than 0.1% epinastine eye drops [24, 26]. Epinastine 0.5% eyelid cream has received approval in Japan to treat and prevent allergic conjunctivitis; the easy-to-use topical formulation may be particularly beneficial in people who find eye drop administration burdensome, and for those with dexterity difficulties [24, 27].

Study limitations included assessment of safety and efficacy only after a single application (i.e. consecutive daily dosing was not evaluated), subjective outcomes (e.g. ease of application, cosmetic acceptability) were not considered, and 0.5% epinastine topical eyelid cream was not directly compared with anti-allergic eye drops that currently represent the standard of care [1, 2, 8–10]. These limitations may be addressed by future studies including the ongoing phase 3 environmental trial, which will evaluate 0.5% epinastine topical eyelid cream, applied once daily for 8 weeks, in approximately 180 patients with allergic conjunctivitis [26].

A key strength of this study is that it was the first to evaluate a once-daily, topical ophthalmic cream (containing a known anti-allergy agent, epinastine) in a population that represents the most prevalent form of allergic conjunctivitis in Japan [5]. Although previous studies have assessed the efficacy of once-daily olopatadine [28], alcaftadine [29] and bilastine [30] eye drops in patients with allergic conjunctivitis, these treatment options are not currently available in Japan. In addition, some of these studies evaluated effectiveness using 16-hour CAC tests [28–33], suggesting that such agents may not provide full 24-hour symptom control (e.g. during sleep). Regardless, the difficulty and discomfort of administering eye drops may lead to poor instillation techniques, suboptimal or missed doses, increased risk of infection and reduced therapeutic benefit [14, 15, 34]; all of which could be avoided by a topically-administered ophthalmic cream that can be easily applied to the eyelids. Thus, in conjunction with its relative ease of application, the results of the current study suggest that 0.5% epinastine topical eyelid cream is a convenient, safe, and effective treatment option for patients with simulated allergic conjunctivitis.

In conclusion, the primary endpoint of reduced ocular itching and conjunctival hyperaemia severity was achieved in this phase 3 study. Superiority of 0.5% epinastine topical eyelid cream was demonstrated over the placebo cream up to 24 h after applying 0.5% epinastine topical eyelid cream to the outer skin of the upper and lower eyelids, suggesting that it is suitable for once-daily use in patients with seasonal allergic conjunctivitis. Moreover, 0.5% epinastine topical eyelid cream displayed an acceptable safety profile in the context of this study, with no AEs, ADRs or clinically relevant changes in ocular assessments reported. Together, these data suggest that 0.5% epinastine topical eyelid cream, via its novel route of administration, may address an unmet clinical need for a convenient, easy-to-use, once-daily anti-allergy medication for the long-term prevention of allergic conjunctivitis. Future studies are recommended to develop epinastine 0.5% ophthalmic cream further.
